# Long-term variability in sugarcane bagasse feedstock compositional methods: sources and magnitude of analytical variability

**DOI:** 10.1186/s13068-016-0621-z

**Published:** 2016-10-18

**Authors:** David W. Templeton, Justin B. Sluiter, Amie Sluiter, Courtney Payne, David P. Crocker, Ling Tao, Ed. Wolfrum

**Affiliations:** National Bioenergy Center, National Renewable Energy Laboratory, 15013 Denver West Pkwy., Golden, CO 80401-3393 USA

**Keywords:** Compositional analysis, Sugarcane bagasse, Variability, Biofuels, NIST RM 8491, MESP

## Abstract

**Background:**

In an effort to find economical, carbon-neutral transportation fuels, biomass feedstock compositional analysis methods are used to monitor, compare, and improve biofuel conversion processes. These methods are empirical, and the analytical variability seen in the feedstock compositional data propagates into variability in the conversion yields, component balances, mass balances, and ultimately the minimum ethanol selling price (MESP). We report the average composition and standard deviations of 119 individually extracted National Institute of Standards and Technology (NIST) bagasse [Reference Material (RM) 8491] run by seven analysts over 7 years. Two additional datasets, using bulk-extracted bagasse (containing 58 and 291 replicates each), were examined to separate out the effects of batch, analyst, sugar recovery standard calculation method, and extractions from the total analytical variability seen in the individually extracted dataset. We believe this is the world’s largest NIST bagasse compositional analysis dataset and it provides unique insight into the long-term analytical variability. Understanding the long-term variability of the feedstock analysis will help determine the minimum difference that can be detected in yield, mass balance, and efficiency calculations.

**Results:**

The long-term data show consistent bagasse component values through time and by different analysts. This suggests that the standard compositional analysis methods were performed consistently and that the bagasse RM itself remained unchanged during this time period. The long-term variability seen here is generally higher than short-term variabilities. It is worth noting that the effect of short-term or long-term feedstock compositional variability on MESP is small, about $0.03 per gallon.

**Conclusions:**

The long-term analysis variabilities reported here are plausible minimum values for these methods, though not necessarily average or expected variabilities. We must emphasize the importance of training and good analytical procedures needed to generate this data. When combined with a robust QA/QC oversight protocol, these empirical methods can be relied upon to generate high-quality data over a long period of time.

**Electronic supplementary material:**

The online version of this article (doi:10.1186/s13068-016-0621-z) contains supplementary material, which is available to authorized users.

## Background

Lignocellulosic biomass has been identified as a potential feedstock for the production of liquid biofuels [1–5]. Such non-edible plant matter can become a carbon-neutral, renewable source of transportation fuel [6], if processes to convert biomass to biofuels can be made economical at the scale of the transportation fuel market [7]. A critical component of NREL’s biofuels program [8, 9] is a robust techno economic analysis (TEA) capability, which allows for the economic comparisons of different conversion processes, the setting of technical goals to achieve cost targets, and determining the cost effects of integrating different processes into a combined process. Challenges to economic biofuel conversions include: identification and production of renewable biomass sources [10–13]; collecting and transporting diffuse sources of biomass to a central depot or conversion facility [14, 15]; the type and mass fraction of components in lignocellulose that can be converted to fuels [16, 17]; capital costs and efficiency of biomass pretreatments [18, 19]; costs and yields from enzymatic hydrolysis [20]; and configuration, yields, scale and efficiency of fermentation [21].

When comparing different feedstocks, process intermediate compositions, or TEA results, it becomes critical to know if any differences seen are significant. It is important to determine the effect of long-term analytical variability on the downstream values derived from this data. One important input into TEA models is the biomass feedstock composition. These biomass compositional methods are empirical, and small differences in the analytical technique can have large effects on the feedstock compositions and therefore the mass balances, conversion yields, and TEA results that are derived from these values.

At NREL, we analyze a NIST RM along with each batch of 6–10 biomass samples as a method verification standard (MVS) to confirm the stability of the analytical conditions. The analysis of the NIST bagasse, run as an MVS for many sample batches, can be used to estimate the method analytical variability over time and its effect on MESP. A short-term (about 6 weeks) study of the analytical variability on a specially prepared corn stover along with NIST bagasse run in two laboratories, by seven analysts has been previously reported [22]. The corn stover variability from this short-term study was used to estimate the effect of primary measurements on MESP [23].

Different sources of analytical variability can be assessed using these replicated NIST bagasse datasets, which include within and between analytical batch variability; and between analyst variability, short-term variability, long-term variability (the subject of this paper), and interlaboratory variability. Understanding the long-term variability will help determine the minimum difference that can be detected in yield, mass balance, and MESP calculations.

Here we report the long-term analytical variability of replicated NIST bagasse analyses based on compositional data, where each sample was individually extracted from seven analysts, two NREL labs, and 119 batches collected over 7 years. We also report on two other sets of bagasse data which can highlight different sources of analytical variability seen in the individually extracted data. Together, these datasets (perhaps the world’s largest NIST bagasse compositional analysis dataset) allow for estimates of the long-term analytical variability. We compare these data with previously published data, and calculate the effects of the variability on MESP values.

## Methods

### NIST bagasse material

The NIST bagasse (RM 8491), which is available for purchase, was collected, homogenized, and analyzed in a round-robin study as described previously [24–26]. The reference compositions for the four NIST biomass RMs were recently re-standardized using an interlaboratory study to update the reported values, and this work showed that the RM compositions had not changed since the original analysis [27]. We run a RM with each analytical batch and it is chosen to most closely match the biomass type being analyzed. We use NIST bagasse for the analysis of herbaceous biomass samples, and it is assumed that the RM behaves similarly to the samples during analysis. The compositional results from the MVS are used as part of a QA/QC protocol (described below) to help determine if the batch results are reported or rerun.

### Compositional analysis methods

Feedstock compositions are determined by a series of solvent extractions, gravimetric analyses, acid hydrolyses, and chromatographic methods to summatively measure the different components of biomass. A history and detailed description of these compositional analysis methods has been described previously [28]. Two different biomass sample types, feedstocks, and solid process intermediate samples (e.g., after pretreatment, enzymatic hydrolysis, or fermentation) are typically analyzed in our lab. These two sample types are prepared differently for compositional analysis, with feedstock samples being extracted and process intermediate samples analyzed without extraction.

For herbaceous feedstocks, ~3 g of NIST bagasse material is extracted and quantified along with the associated feedstock samples. For process intermediate samples, some large batches (~500 g) are bulk extracted, and in these cases the total amount of extractives removed are not quantified. Both extracted feedstocks and unextracted process intermediate samples are hydrolyzed for 1 h in 72 % H_2_SO_4_ followed by dilution to 4 % acid, and a secondary hydrolysis for 1 h at 121 °C in an autoclave. This two-stage hydrolysis breaks down the structural sugars (cellulose and hemicellulose) to monomers for HPLC detection. The concentration of monomer carbohydrates is converted to an anhydro basis for reporting purposes. For instance, the glucose measured in the hydrolysate is reported as the polymer form glucan irrespective of the source of glucose (cellulose, hemicellulose, etc.), since it is the polymer form of the carbohydrate found in the sample. This is true for galactose and arabinose as well; these monomers are likely present as side chains of the hemicellulose. The remaining solids are measured gravimetrically as lignin or more specifically as acid insoluble residue (AIR), also known as Klason lignin. A UV–Vis analysis is used to measure acid-soluble lignin (ASL) in the hydrolysate liquor, and these values are either reported separately or combined to report total lignin.

### QA/QC protocols

We use a combination of experienced analysts, extensive training, proper analytical technique, and QA/QC oversight to generate the analytical variability reported here. This QA/QC protocol uses the results from the batch MVS compared to known values, the component closures of the samples and MVS, replication among analytical duplicates, and specific markers within the HPLC data to determine if the results are reliable. A set of QA/QC parameters, including means and tolerances established from external publications and internal studies, has been developed to evaluate data batches. Deviations from the QA/QC parameters for a sample are sufficient to warrant repeated analysis of that individual sample. Unexplained deviations within the MVS analysis lead to reanalysis of the entire batch of samples. Repeated excursions beyond the MVS limits by a single analyst triggers closer technique scrutiny or even retraining. Thus, the data presented in this manuscript include only those samples that passed internal QA/QC checks.

### Calculation spreadsheets

Determining summative mass closure on a compositional batch requires hundreds of measurements and calculations. NREL has published Microsoft Excel workbooks that perform all of the necessary calculations, along with flags to identify samples that do not replicate within specified uncertainties. They have proven to be a useful tool to the biofuels community, as mistakes in calculations can be difficult to detect. These workbooks can be downloaded from NREL’s biomass website (http://www.nrel.gov/bioenergy/biomass-compositional-analysis.html).

For this work, two workbooks were employed: one for feedstocks (which include extraction) and one for process intermediate samples (which do not require extraction). The feedstock workbook mathematically corrects all structural (or non-extractable) material by the amount of extractives removed. Thus, the structural values will decrease, as they are put on a whole (including extractives) dry weight basis. In comparison, process intermediate samples that are analyzed without correcting for extractives content will only report structural components (i.e., reporting on an extractives-free basis). Both routes of analysis should result in a mass closure close to 100 % component closure (though on different reporting bases), which suggest that all the biomass components are being detected and counted properly. Both workbooks calculate carbohydrate values after correcting for losses during hydrolysis using a SRS run with each batch. It is also possible to back calculate this data using an average SRS value, in an attempt to remove this possible source of variability.

### Scientific data management system

Data from experimental batches were recorded, calculated, and analyzed in Microsoft Excel spreadsheets, though they did not provide a convenient method for data management nor for meta-analysis of large datasets. To resolve these issues, batch spreadsheets were collected in a web-enabled, in-house scientific data management system built with open source tools including Oracle’s Java and MySQL, Google’s Google Web Toolkit, Red Hat’s JBoss and Hibernate, and Apache POI. The system tracked sample data, work records, and analytical results, and archived copies of related files. Data mining was facilitated by a simple, web-based interface. Selected compositional data were collected and downloaded in Microsoft Excel file format to be curated further and analyzed.

### Datasets analyzed

There are three datasets analyzed for this paper which are presented in increasing method complexity order as follows:

1. The *short*-*term round*-*robin set* (ST-RR) includes 67 samples from a single bulk extraction batch of NIST bagasse material run in batches of 8–12 bagasse replicates by seven different analysts over 2 months, though no analyst ran multiple batches. These samples were analyzed in a dedicated experiment designed to control and minimize many common sources of analytical variability. All samples were taken from one bulk extraction and were analyzed in a single laboratory using the same autoclave, and on the same HPLC. Analytical batches contained only the NIST bagasse material to allow examination of within batch variability.
2. The *long*-*term extractives-free dataset* (LT-EF) includes 295 samples from several bulk-extracted NIST bagasse batches that were analyzed in duplicate and included as MVS along with batches of process intermediate samples. The extractives-free results from this dataset include variability resulting from seven analysts in two labs using multiple autoclaves, HPLC systems, and analytical standards over 7 years. This dataset does not include analytical uncertainties due to individual extractions.
3. The *long*-*term individually extracted biomass dataset* (LT-IE) includes 119 samples from individually extracted bagasse run by seven analysts in two labs over 7 years. In addition to the variability sources described in the LT-EF set, this dataset includes analytical variabilities due to extractions.

### Statistics

Compositional data from the calculation spreadsheets were aggregated into a text file (Additional file 1) for statistical analysis using “R” statistical software [29] (analysis code provided as Additional file 2) . We used P < 0.05 as a level for tests of significance. Given that there are dozens to hundreds of replicates in these datasets, there is the power to detect statistically significant yet practically small differences. Compositional differences of less than 0.3 % dry weight are not considered practically significant.

### TEA analysis

TEA analysis includes a conceptual level of process design to develop a detailed process flow diagram (based on research data); rigorous material and energy balance calculations (via a commercial simulation tool, Aspen Plus); capital and project cost estimations (via an economic model using spreadsheets); a discounted cash flow economic model; and the calculation of an MESP for an “Nth” plant. The 2011 NREL biochemical cellulosic ethanol design case model [9] is used as the benchmark model for this study. Variation in the feedstock composition (principally the structural carbohydrates) not only impacts the overall process design, but can impact the ethanol yield and MESP [30]. Thus, we have performed a sensitivity analysis using the benchmark model, and we performed MESP calculations on the bagasse compositions from the LT-IE dataset. All other model parameters and the overall conceptual process design are kept constant; and we report the variability in MESP based only on compositional variability.

## Results and discussion

### Short-term round-robin bagasse data

**Fig. 1:**
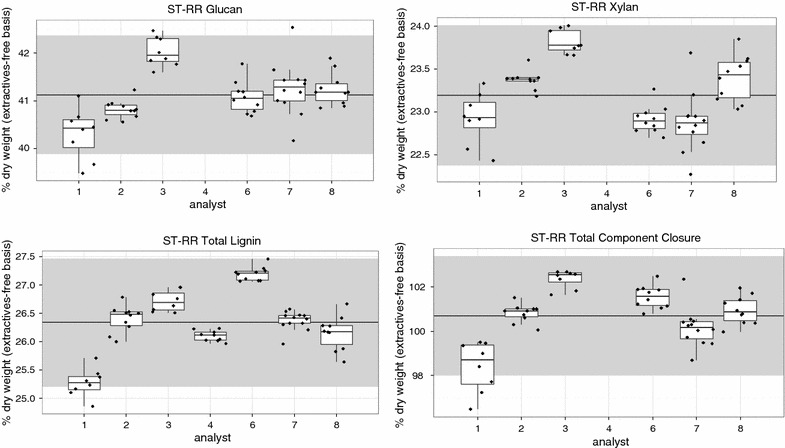
Compositional data from *short*-*term round*-*robin set* (RR) plotted by analyst. Each batch was run by a different analyst with 7–10 replicates of the NIST RM 8491 material. This material was extracted in bulk, and all the data were collected from one chromatography system in order to minimize variability. The *gray band* in the background shows the two times the grand standard deviation centered on the grand average (denoted by the *central line*) for the entire RR dataset. Analysts 5 and 9 did not run this experiment. The carbohydrate data for analyst 4 was an outlier and not included here, therefore a total component closure cannot be calculated

A general statistical summary of the short-term round-robin (ST-RR) set is shown in Table 1. The data reported here on an extractives-free basis are artificially inflated compared to previous bagasse data reported on an as-received basis, although the standard deviations for the major components are similar [22]. A pooled standard deviation is provided that minimizes the contribution of the batch-to-batch variability and is seen to be universally lower than the regular standard deviation for each constituent. This can be seen in Fig. 1, where each batch has variances that are tighter than that of the complete sample set, and the overall variability is driven by the batch-to-batch variability.

**Table 1:** The *short*-*term round*-*robin set:* descriptive statistics of NIST RM 8491 sugarcane bagasse composition on an extractives-free, % dry mass basis

	Ash	ASL	AIR	Glucan	Xylan	Galactan	Arabinan	Acetyl	Total component closure
Mean	3.71	4.18	22.16	41.13	23.19	0.76	1.84	3.34	100.71
SD	0.20	0.56	0.34	0.62	0.41	0.19	0.24	0.05	1.34
Pooled SD	0.15	0.09	0.19	0.40	0.24	0.09	0.13	0.04	0.70
N	67	66	66	58	58	55	58	67	58
RSD (%)	5.4	13.3	1.5	1.5	1.8	24.9	13.1	1.4	1.3
RpSD (%)	4.1	2.1	0.9	1.0	1.0	12.0	7.1	1.3	0.7

These samples were taken from a single large-scale extraction and analyzed in replicate (7–10 times) per batch/analyst. They were analyzed in one lab on one HPLC system using the same standards as part of an experiment to artificially reduce variability. The pooled standard deviation was calculated by batch/analyst

*SD* standard deviation, *Pooled SD* pooled standard deviation, *N* number of samples analyzed, *RSD* relative standard deviation, *RpSD* relative standard deviation calculated from the pooled SD, *ASL* acid-soluble lignin, *AIR* acid insoluble residue

### Long-term mean results

**Fig. 2:**
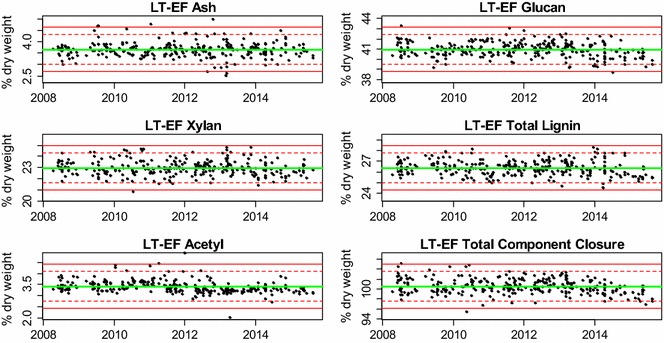
Control charts of compositional data for the *long*-*term extractives-free dataset* (bulk-extracted NIST RM 8491 sugarcane bagasse composition) plotted chronologically. Samples in this set were analyzed along with process intermediate samples. The *central green line* denotes the average value, while the *dashed red lines* show two times the standard deviation and *solid red lines* show three times the standard deviation

**Fig. 3:**
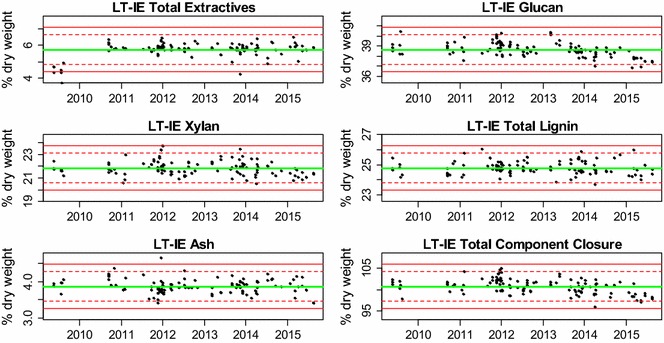
Control charts of compositional data for the *long*-*term individually extracted biomass dataset* (individually extracted NIST RM 8491 sugarcane bagasse composition) plotted chronologically. This set was analyzed along with feedstock samples. The *central green line* denotes the average value, while the *dashed red lines* show two times the standard deviation and *solid red lines* show three times the standard deviation

The mean values for the major bagasse constituents in the LT-EF and LT-IE datasets are presented as control charts in Figs. 2 and 3, respectively. These charts show consistent mean compositional values and standard deviations over 7 years. This suggests that the standard compositional analysis methods used to generate these data were performed consistently, and that the feedstock itself remained constant over this time period. Most of the individual data points fall between the two standard deviation (dashed) lines with a small minority falling outside two or even three standard deviations from the overall mean result. The descriptive statistics for the LT-EF and LT-IE datasets are presented in Tables 2 and 3, respectively. However, on an extractives-free basis, an ANOVA showed that the mean values for glucan, total lignin, and total component closure were the same (data not shown) for all three datasets (ST-RR, LT-EF, and LT-IE) reported here. The ANOVA for xylan showed a statistically significant (though not practically significant) mean difference of 0.22 % between the ST-RR and the LT-EF datasets, which only can be discerned using such large datasets. As opposed to the ST-RR dataset, the pooled standard deviations (by analyst) are essentially the same as the regular standard deviations, which suggests that over time the batch-to-batch variability can even out among the analysts. Even though the means are practically the same, there are some significant differences noted in the standard deviations, which can be used to assign causes for the sources of variability as described below.

**Table 2:** The *long*-*term extractives-free dataset*: descriptive statistics of NIST RM 8491 sugarcane bagasse composition on an extractives-free,  % dry mass basis

	Ash	Protein	ASL	AIR	Glucan	Xylan	Galactan	Arabinan	Acetyl	Total component closure
Mean	3.66	0.55	4.51	21.81	40.96	22.97	0.94	1.94	3.41	100.55
SD	0.33	0.05	0.51	0.53	0.73	0.67	0.32	0.32	0.33	1.50
Pooled SD	0.32	0.03	0.42	0.43	0.74	0.67	0.32	0.33	0.34	1.49
N	288	103	234	233	293	293	292	293	295	291
RSD (%)	8.9	9.8	11.3	2.4	1.8	2.9	34.1	16.7	9.8	1.5
RpSD (%)	8.8	5.5	9.2	2.0	1.8	2.9	34.1	16.9	9.9	1.5

This set includes bulk-extracted bagasse samples run along with process intermediate samples. They were analyzed in different labs and on different HPLC systems

*SD* standard deviation, *Pooled SD* pooled standard deviation, *N* number of samples analyzed, *RSD* relative standard deviation, *RSD* relative standard deviation, *RpSD* relative standard deviation calculated from the pooled SD, *ASL* acid-soluble lignin, *AIR* acid insoluble residue

**Table 3:** The *long*-*term individually extracted biomass dataset:* descriptive statistics of NIST RM 8491 sugarcane bagasse composition on as-received biomass (including individually determined extractives), % dry mass basis

	Ash	Protein	Water extractives	Ethanol extractives	Sucrose	ASL	AIR	Glucan	Xylan	Galactan	Arabinan	Acetyl	Total component closure
Mean	3.87	0.55	3.86	1.87	0.22	4.45	20.35	38.64	21.85	0.87	1.90	3.21	100.78
SD	0.20	0.03	0.43	0.13	0.31	0.53	0.33	0.74	0.63	0.30	0.26	0.38	1.74
Pooled SD	0.19	0.02	0.35	0.13	0.26	0.51	0.32	0.75	0.64	0.30	0.26	0.35	1.75
N	119	48	122	119	119	115	112	119	119	119	119	118	119
RSD (%)	5.3	6.2	11.0	6.9	137.8	11.9	1.6	1.9	2.9	34.9	13.7	11.8	1.7
RpSD (%)	4.9	3.7	9.0	6.9	115.0	11.5	1.6	1.9	2.9	34.6	13.7	10.9	1.7

This set includes bagasse samples run along with feedstock samples. They were analyzed in different labs and on different HPLC systems. This data is used to calculate the long-term variability result

*RSD* relative standard deviation, *SD* standard deviation, *Pooled SD* pooled standard deviation, *N* number of samples analyzed, *RpSD* relative standard deviation calculated from the pooled SD, *ASL* acid-soluble lignin, *AIR* acid insoluble residue

### Mean comparisons to previous bagasse data

The LT-IE mean compositional data agrees well with previously reported data including a short-term set of 13 NIST bagasse samples that were run by seven analysts in two laboratories over the course of several weeks [22]. These compositional values also agree with the re-standardized values reported for the four NIST biomass RMs [27], with the exception of the glucan, galactan, and acid-soluble lignin components. The NIST bagasse composition was re-standardized in a round robin from 11 laboratories (up to 13 replicates) using median statistics, not mean statistics as reported here. The median value and uncertainties reported give less weight to extreme values and are advantageous for smaller or highly variable datasets, as was seen in these interlaboratory results. Not included in this study is the variability seen with these methods between institutes, which we recently demonstrated can be considerable [27].

### Comparing standard deviations to determine the causes of variability

Comparing the component standard deviations (rather than the mean values) from these three datasets (ST-RR, LT-EF, and LT-IE) can reveal the sources of analytical variability, and estimate the relative magnitude of these factors. We can use the following model to assign the different sources of analytical variabilities seen in these datasets, assuming they are additive:

$$\varepsilon_{\text{TOTAL}} \text{ = }\varepsilon_{\text{BATCH}} \text{ + }\varepsilon_{\text{ANALST}} \text{ + }\varepsilon_{\text{SRS}} \text{ + }\varepsilon_{\text{EXTRACTION}} \text{ + }\varepsilon_{{{\text{SHORT}}\;{\text{TERM}}}} \text{ + }\varepsilon_{{{\text{LONG}}\;{\text{TERM}}}} \text{ + }\varepsilon_{\text{OTHER}}.$$

This equation assumes that the total analytical variability is the sum of the variabilities due to the batch-to-batch differences, analyst-to-analyst differences, the effect of using an individual or an overall average SRS value, and extracting or not extracting the biomass. In addition to these, the variabilities in the analysis system (e.g., using multiple HPLCs, multiple batches of analytical standards, and different autoclaves) that occur over a few weeks (short-term) or several years (long-term), along with other unknown other sources, add to the total analytical variability.

Measuring the standard deviation of replicate bagasse runs can determine the combined analytical variability, though not the individual sources that this model would suggest. With these three datasets, the combined standard deviation is the summation of different combinations of individual sources, and by comparing them we can infer the magnitude of individual sources, as seen in the equations below:

$${\text{SD of LT-IE dataset}}\;{ = }\;\varepsilon_{\text{BATCH/ANALST}} + \varepsilon_{\text{SRS}} + \varepsilon_{\text{EXTRACTION}} + \varepsilon_{{{\text{SHORT}}\;{\text{TERM}}}} + \varepsilon_{{{\text{LONG}}\,{\text{TERM}}}} + \varepsilon_{\text{OTHER}}$$

$${\text{SD of LT-EF dataset}}\;{ = }\;\varepsilon_{\text{BATCH/ANALST}} + \varepsilon_{\text{SRS}} + \varepsilon_{{{\text{SHORT}}\;{\text{TERM}}}} + \varepsilon_{{{\text{LONG}}\,{\text{TERM}}}} + \varepsilon_{\text{OTHER}}$$

$${\text{SD of 2010 ST data}}\;{ = }\;\varepsilon_{\text{BATCH/ANALST}} + \varepsilon_{\text{SRS}} + \varepsilon_{{{\text{SHORT}}\;{\text{TERM}}}} + \varepsilon_{\text{OTHER}}$$

$${\text{SD of ST - RR dataset}}\;{ = }\;\varepsilon_{\text{BATCH/ANALST}} + \varepsilon_{\text{SRS}} + \varepsilon_{\text{OTHER}}.$$

Thus, the difference in the standard deviations between the LT-IE and LT-EF sets would be due to the effect of extraction variability, and the difference between the LT-EF and ST-RR would be due to long-term variations in the analysis system. Other comparisons can be made to tease out sources of analytical variability. A comparison of the pooled standard deviation (by analyst) with the regular standard deviation can reveal differences between batch and analyst. For the carbohydrate components, the SRS correction factor can be back calculated using an average SRS value which removes the variability due to individual SRS determinations.

**Fig. 4:**
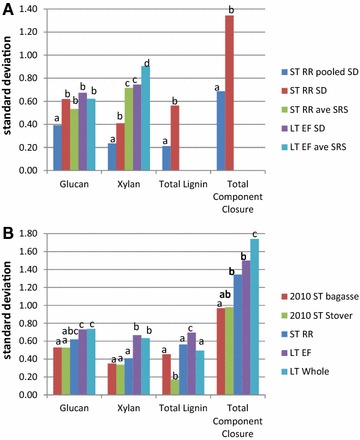
Comparison of standard deviations calculated on different datasets.** A** shows comparisons of short- and long-term variability between pooled, regular, and calculated based on average sugar recovery values of standard deviations.** B** shows differences in regular standard deviation between short- and long-term data sets and includes previously analyzed data. *Lower case letters* indicate significant differences using* F* test

Figure 4A shows the comparison of the standard deviations of the three largest components plus total component closure as determined on different sets of biomass. In Fig. 4A, the first two bars for each component shows the pooled and regular standard deviations for the ST-RR dataset. Each batch was run by a different analyst, so it is not possible to separate batch and analyst effects. The pooled standard deviation measures the variability within the batch/analyst for this set, and the regular standard deviation measures the between batch/analyst variability. This can be seen in Fig. 1, where the vertical deviations are similar across the analysts while the differences between analyst values are larger.

The pooled standard deviation from the RR dataset shows a minimum short-term analytical variability for these methods after attempting to artificially reduce common sources of variability, by restricting the analysis to one extracted batch of bagasse run in one autoclave and on one HPLC. The regular standard deviation is significantly higher (at least P < 0.002) compared to the corresponding pooled standard deviation for all the major components, which suggests that the analyst/batch variability is a significant driver of the overall variability. This finding corresponds with previous corn stover and bagasse ST experiments showing similar effects of analyst/batch on these methods [22].

**Fig. 5:**
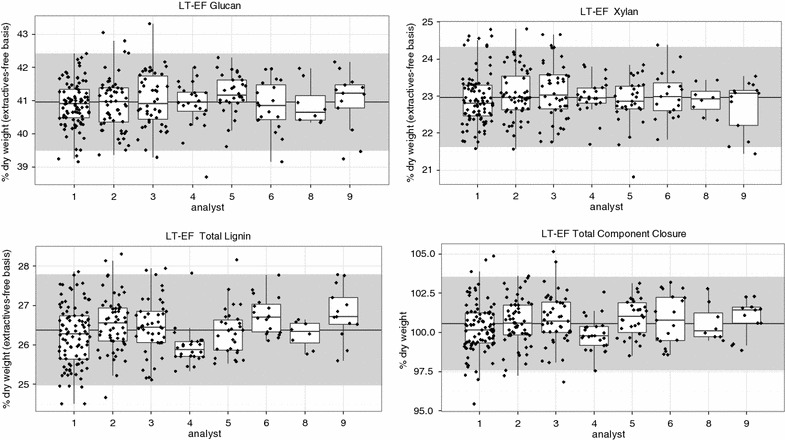
*Box plot* of major components presented by analyst on LT-EF sample group. The *gray band* in the background shows the two times the standard deviation centered on the mean (denoted by the *central line*) value for the entire set. Analyst 7 did not run this sample type

While this ST-RR data cannot differentiate between batch and analyst, the long-term datasets show mainly consistent compositions among the different analysts. Figure 5 shows the average compositions of the major components from the LT-EF dataset presented by analyst. A Turkey honest significant difference analysis shows that there is no difference in the mean compositional values for the LT-EF glucan and xylan along with all major components in the LT-IE dataset (data not shown). Thus, the variability seen by each analyst is due to different batches, although the average results are similar among all analysts. Analyst number 4 has values statistically (though not practically) lower compared to the other analysts, for the LT-EF total lignin and therefore total component closure. In general, these data suggest that analyst-to-analyst variability is not a significant factor and that batch-to-batch variability is a large contributor in total variability. More effort is needed to identify and reduce the sources of batch-to-batch variability seen here such as volume losses during lignin separation, autoclave heating differences, or other effects.

For the major carbohydrate components (glucan and xylan), it is possible to separate the effect of different SRS calculation methods on the component concentration. The last four bars in Fig. 4A for glucan and xylan show the SD calculated normally with corresponding SRS run at the same time as the standards and the same data calculated with the average SRS response calculated from the entire time period. The mean values did not change for the components, though some of the SD did change. For glucan, the average SRS values were not statistically different, and the xylan values were statistically higher (and therefore worse) for the average SRS. The combination of similar glucan values and worse xylan values for the averaged SRS data suggests the individual SRS is making appropriate adjustments to the final compositional value, and using an average SRS value would add unnecessary variance to the xylan value.

Figure 4B shows the differences between short-term and long-term variabilities for the major components in biomass. In general, the long-term variability is higher compared to the short-term variability, for both the 2010 ST data and the ST-RR data. For all the major components, the LT-IE variability is significantly higher compared to the previously reported 2010 ST Stover variability, which was used to determine MESP variability from primary measurements [31]. The increase in variability from short-term to long-term could be due to different lots of HPLC standards, standard concentration changes over time, changes to the HPLC columns and systems, environmental changes in the lab, and the accumulation of other unknown causes. Even with higher long-term variability, these values represent plausible minimum variabilities when applying our QC methods, although it is possible to get much higher variabilities than reported here.

### Effects of long-term variability on MESP

**Fig. 6:**
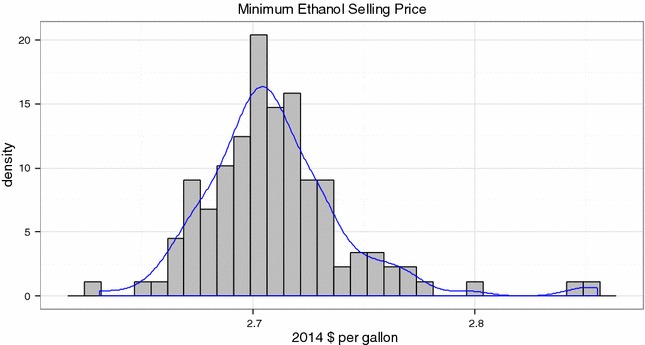
Histogram of MESP values calculated based on 2011 biochemical design case model using complete LT-IE bagasse compositions, which shows variation due to feedstock composition variability. Average MESP = $2.71 per gallon with a standard deviation of $0.03 per gallon

The long-term variability determined here propagates into variability in the price of ethanol from a biorefinery or MESP. In order to understand the economic effect of the long-term variability seen in NIST bagasse on MESP, we inputted the compositions of the individually extracted NIST bagasse into the 2011 biochemical design case model [9]. The LT-IE dataset is the most complete source of analytical variability since it includes all sources of variability discussed previously. Figure 6 shows the histogram of the MESP calculated from the LT-IE dataset, which shows the average MESP at $2.71 /gal with standard deviation of $0.03 /gal (in 2014$). Thus, when feedstock compositional analysis variability is well controlled, the effect on MESP is small.

Previous work provided an estimate of the effect of *primary measurements* (composition *plus* mass and volumetric flow estimates) on MESP, which showed a MESP of $2.21 with a standard deviation of $0.15 gal (in 2007$) [30]. This estimate was based on short-term variability, which is lower compared to the long-term variability presented here. The feedstock portion of the primary measurement variability was estimated to be 6.7 % of the total or $0.01 /gal (in 2007$) [22]. Thus, the variability in determining mass and volume flows through the biofuels process drives most of the MESP variability from primary measurements rather than the feedstock analytical variability. Even though the long-term variability determined here is higher compared to the short-term variability determined previously, the effect on MESP is small. Previous estimates of the effect of different *sources of corn stover* showed an MESP of $2.20 gal with a standard deviation of $0.07 /gal (in 2007$) [30]. Taken together, this shows that the long-term analytical variability does not limit the ability to determine differences between conversion processes or between different sources of feedstock.

## Conclusions

Here we report the long-term feedstock analytical variability for NIST bagasse compositional analysis results run over 7 years. This includes long-term effects such as HPLC instrument drift, different standard sets, and seasonal changes. This long-term analytical variability data can be used as a guide to determine if compositional differences are significant. The long-term analytical variability is higher compared to previously determined short-term analytical variability, although neither of these sources drove the MESP variability estimate.

The analytical variability reported here is not the average variability seen when utilizing these methods (especially when first starting to use these methods), but represent the inherent variability that can be expected with highly trained analysts using these empirical methods regularly. Certainly, smaller numbers of replicates run over short periods of time may lead to lower variabilities than those reported here, but are not necessarily better or different than those reported here. The data we present in this work demonstrate that it is possible for a team of analysts to obtain consistent results over time, not that it is necessarily easy.

## Additional files

**Additional file 1.** Comma separated value (.csv) file containing bagasse compositional value data used in this analysis.

**Additional file 2.** “R” code used to analyze data file.

## Abbreviations

LT-EF: long-term extractives-free dataset
LT-IE: long-term individually extracted dataset
MESP: minimum ethanol selling price
MVS: method verification standard
NIST: National Institutes for Science and Technology
NREL: National Renewable Energy Laboratory
RM: reference material
ST-RR: short-term round-robin dataset
SRS: sugar recovery standard
TEA: technoeconomic analysis
